# Flexible and frequency reconfigurable CPW-fed monopole antenna with frequency selective surface for IoT applications

**DOI:** 10.1038/s41598-023-34917-y

**Published:** 2023-05-24

**Authors:** Ahmed A. Ibrahim, Hesham. A. Mohamed, Mahmoud A. Abdelghany, Emad Tammam

**Affiliations:** 1 Electronics and Communications Engineering Department, Minia University, Minia, 61111 Egypt; 2 Electronics Research Institute (ERI), Joseph Tito St, Huckstep, El Nozha, Cairo, 11843 Egypt; 3 Electrical Engineering Department, College of Engineering, Prince Sattam Bin Abdulaziz University, 11991 Wadi Addwasir, Saudi Arabia

**Keywords:** Engineering, Electrical and electronic engineering

## Abstract

This paper proposes a flexible, frequency-reconfigurable monopole antenna design with frequency selective surface (FSS) for Internet of Things (IoT) applications. The proposed antenna operates at three of the IoT frequency bands. This antenna is a coplanar waveguide (CPW)-fed monopole with two balanced arms printed on a thin ROGERS 3003 flexible substrate. The length of the right-hand arm of the antenna is used to achieve frequency reconfiguration by using PIN diodes. Three frequency modes of operation have been obtained; the 2.4 GHz frequency band with the right-hand arm is fully truncated, the 3.5 GHz frequency band with the two arms is completely maintained, and the 4 GHz frequency band with the right-hand arm is partially truncated. To improve the gain of the antenna, a simple FSS surface is designed to be placed under the antenna at a distance of 15 mm. The FSS operates efficiently from 2 to 4.5 GHz and has improved the gain of the antenna. A maximum gain of 6.5 dBi, 7.52 dBi, and 7.91 dBi has been achieved at the three frequency bands respectively. The behavior of the flexible antenna has been evaluated in both the flat and bent states, and stable performance has been observed in both cases.

## Introduction

Recently, IoT has become an evolutional technology that aims to ease communication between human beings and their surrounding environment. The IoT merges the human and his surrounding wireless devices and sensors in a single network in real-time^1–4^. The IoT technology put big challenges on the working communications systems as a huge amount of data is required to be transferred between the wireless nodes connected to the network^5^. By the way, the tremendous speed of progress in wireless communications techniques has led to an urgent necessity to design a high-speed system with efficient spectrum utilization. This in turn has led to the possibility of exploiting a single wireless device in more than one application, and then more than one frequency band has to be utilized. The antenna is characterized by its large size with respect to other components, and it will be impractical to design an antenna for each frequency band on the same platform. For these reasons, a careful design of an antenna that can satisfy the requirements of the next IoT technologies is a vital necessity^4^. One of the successful choices that satisfy the requirements of IoT applications is the reconfigurable antennas^6–8^. Reconfigurable antennas can change their behavior dynamically in a controlled fashion, i.e., they can change their characteristics such as operating frequency, radiation pattern, and polarization^9,10^. To achieve the required control on the antenna, a high-frequency switching device such as a varactor diode, PIN diode, field effect transistor (FET), or micro-electromechanical systems (MEMS) can be utilized^11,12^. With each switching process, the electrical equivalent circuit of the reconfigurable antenna changes, providing different behavior in each case.

Due to their interesting characteristics, reconfigurable antennas have attained more attention from researchers. The reconfigurability of the antenna can be applied to any of its performance parameters such as operating frequency band, radiation characteristics, or polarization.

Some reconfigurable antennas have been designed with frequency reconfigurability^13–15^. In^7^, a compact reconfigurable microstrip antenna has been designed to switch between different frequency bands which include many of the wireless standards such as ZigBee, WiMAX, Bluetooth, and GSM. Some research papers have reported reconfigurable multiband antenna while more than one frequency band can be switched simultaneously^6,16,17^. In^18^, a dual-band reconfigurable multiple input multiple outputs (MIMO) antenna has been proposed to cover the frequency band 1.3–2.6 GHz. Frequency reconfiguration has been realized by a varactor diode on the microstrip feed line. The antenna has achieved dual-band operation in each switching state. The reconfigurable antenna can be designed with radiation pattern reconfigurability, i.e., the radiated power distribution can be controlled spatially in the antenna's surrounding area^19–21^. In^19^, a reconfigurable radiation pattern MIMO antenna is designed as a rectangular monopole with two parasitic strips equipped with PIN diodes. According to the suitable operation switching between the two strips they act as a reflector or director and then the radiation pattern is switched between directional and bidirectional modes. Moreover, the reconfiguration of the antenna can be applied to the polarization of the radiated fields^22–26^. In this case, the antenna can be reconfigured to switch between more than one polarization pattern. For example, a reconfigurable polarization antenna has been proposed in^27^.

In the case of some IoT nodes that are related to health care and biomedical applications where the flexibility of the antenna is the main requirement^20,28,29^. Design of the reconfigurable antenna on a flexible substrate is a challenging process due to the difficulties associated with the integration of the switches to a flexible surface in addition to the associated mechanical stability and electronic robustness issues. Despite the large number of reconfigurable antennas that appeared in the literature, a small ratio of them have been printed on flexible substrates.

In this paper, a frequency reconfigurable antenna printed on a flexible substrate and equipped with FSS is presented. The antenna is a two arms planar monopole fed by a CPW feed line and matched to 50 Ω impedance. Two PIN diodes are used to split one of the two arms of the monopole into three parts, and accordingly, three frequency bands of operation are obtained. The antenna is printed on a ROGERS 3003 flexible substrate and its performance is tested under different degrees of bending. To improve the gain of the antenna, a wideband FSS is designed to be placed under the antenna. The designed FSS works as an upward reflector over the whole antenna's operating frequency. The following section of the paper discusses the reconfigurable antenna design process. The antenna description is studied in “Antenna description and design”. “Results and discussions” investigated the performance of the reconfigurable antenna in the flat and bent positions. The FSS operation is presented, and is followed by the behavior of the reconfigurable antenna with FSS is discussed. In “Conclusion”, a conclusion of the main points comes at the end of the paper.

## Antenna description and design

**Figure 1:**
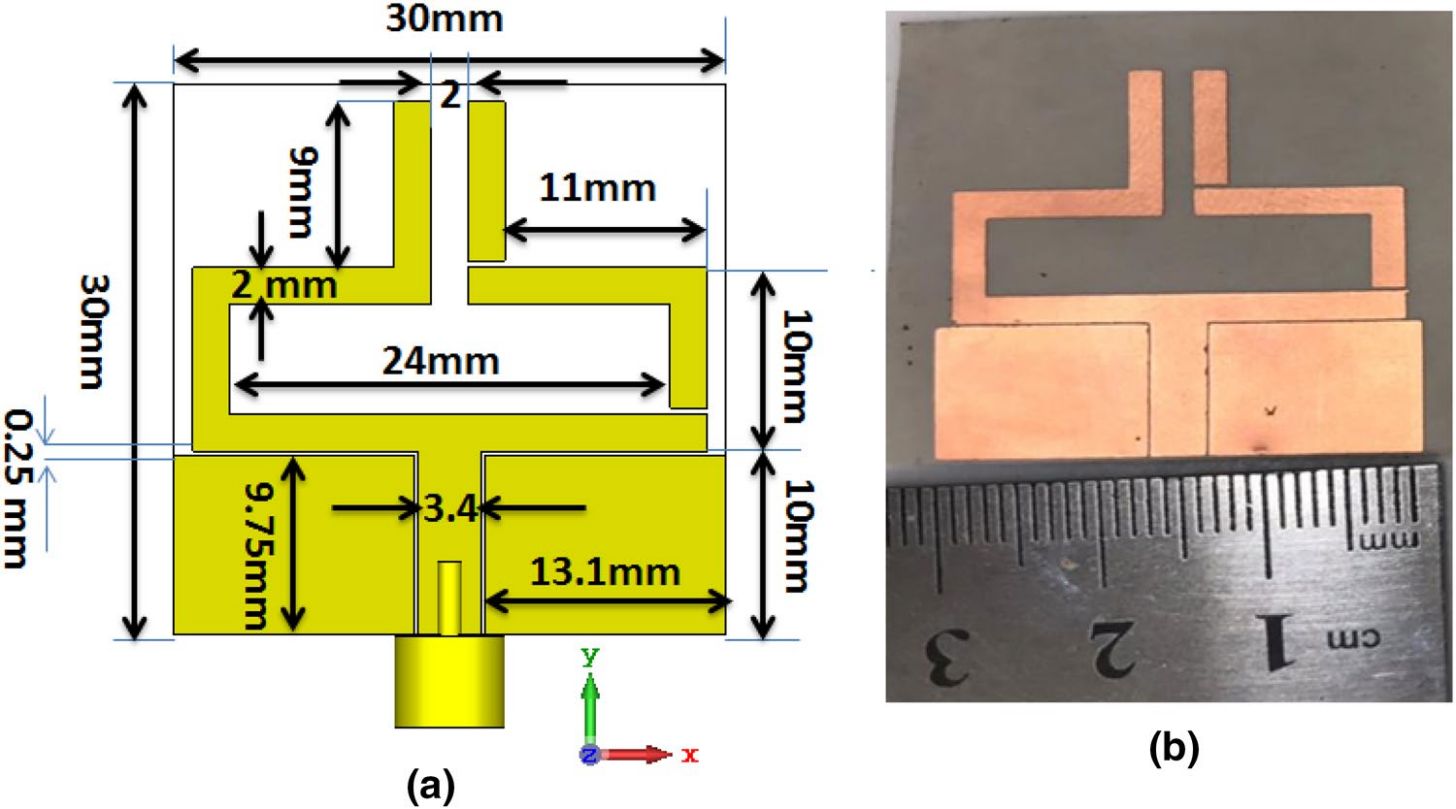
Configuration of the proposed antenna, (**a**) simulated antenna, (**b**) fabricated antenna.

**Figure 2:**
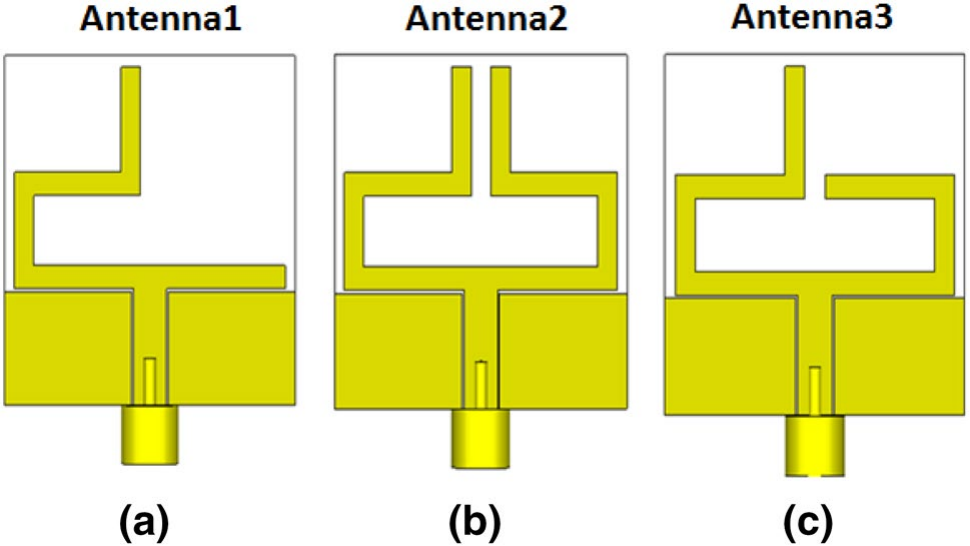
Equivalent antenna structures of the three modes of operation.

The proposed antenna's radiator is constructed as a CPW-fed monopole with a width of 3.4 mm and two gaps of 0.2 mm to achieve the 50 Ω and with two balanced arms as illustrated in Fig. 1. A flexible ROGERS 3003 substrate with ε_r_ = 3, h = 0.13 mm, and tangent loss tan δ = 0.001 is utilized. The antenna dimensions are optimized as displayed in Fig. 1a while the overall size of the antenna is 30 × 30 × 0.13 mm^3^. The reconfigurability of the antenna is achieved by splitting the right-hand arm into three parts while two PIN diodes are used to interconnect these three parts. The state of each PIN diode ON or OFF decides the connection or disconnection of the split parts. The photograph of the fabricated antenna with the disconnecting slots of the right-hand arm is shown in Fig. 1b. Reconfiguration of the antenna results in three pertains modes. Simulation development of the antenna is displayed in Fig. 2. The first mode of operation is seen in Fig. 2a. Antenna 1 is constructed by removing the right-hand arm completely and maintaining the other arm to operate at 2.4 GHz. In the second mode of operation, Antenna 2 displayed in Fig. 2b is the complete reference antenna and it works at 3.5 GHz. The third mode of operation involves the construction of Antenna 3 by removing the upper part of the right-hand arm as shown in Fig. 2c. Shortening of the right-hand arm has resulted in shifting the operating frequency band upwards to the 4 GHz bands.

## Results and discussions

**Figure 3:**
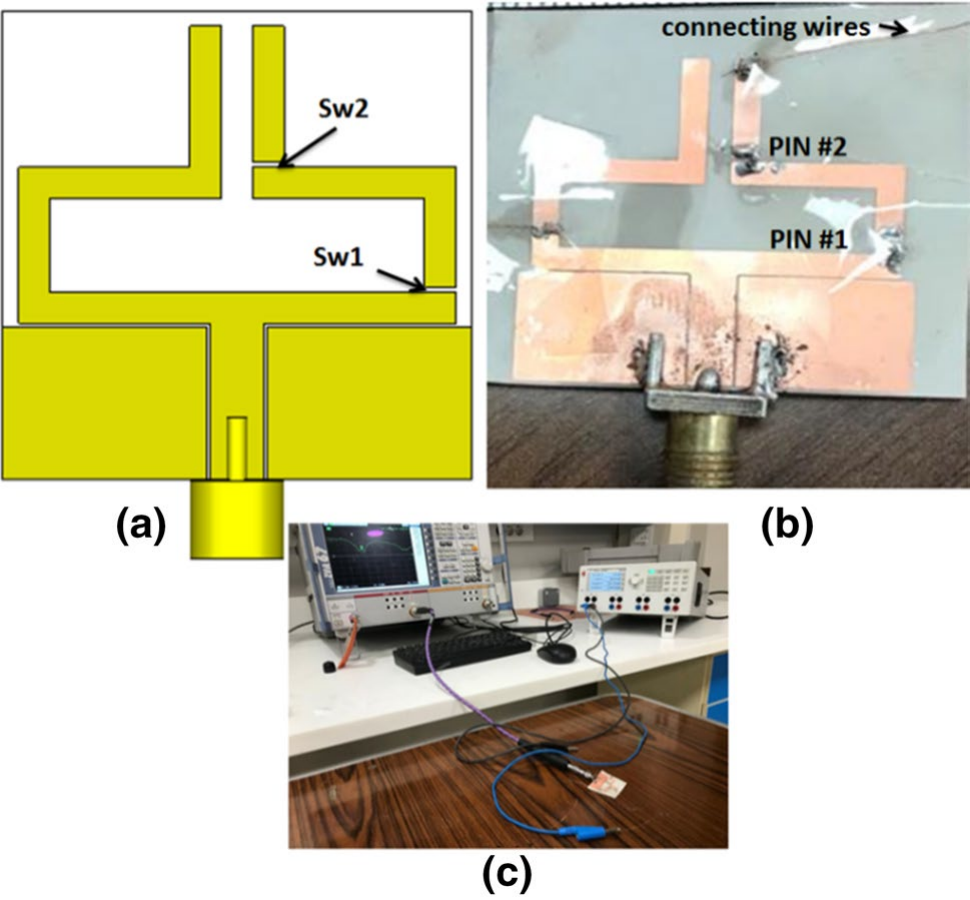
Antenna configuration; (**a**) schematic photo, (**b**) photograph of the fabricated antenna, (**c**) measurement setup of the antenna.

The reconfigurable antenna has been simulated using CST Microwave Studio Software. Fabrication and measurements have been conducted in the Electronics Research Institute (ERI) Labs. To be compromised with the requirements of IoT applications that require a changeable position of the antenna, the antenna has been added on a flexible substrate. The antenna has been fabricated using a chemical etching process (photo-lithographic technique). The schematic of the antenna is illustrated in Fig. 3a. The two PIN diode switch positions are shown in the figure. Fig. 3b displays the fabricated photo of the antenna. The actual PIN diodes in addition to the soldered biasing wires are shown in the figure. A vector network analyzer (model Rohde & Schwarz ZVA 67) is utilized to test the fabricated antenna's electrical behavior. Fig. 3c illustrates the antenna measurement setup while a DC power source is used for biasing the PIN diodes and a VNA is used for the measurements of the return loss.

### A. Antenna's electrical behavior 

**Figure 4:**
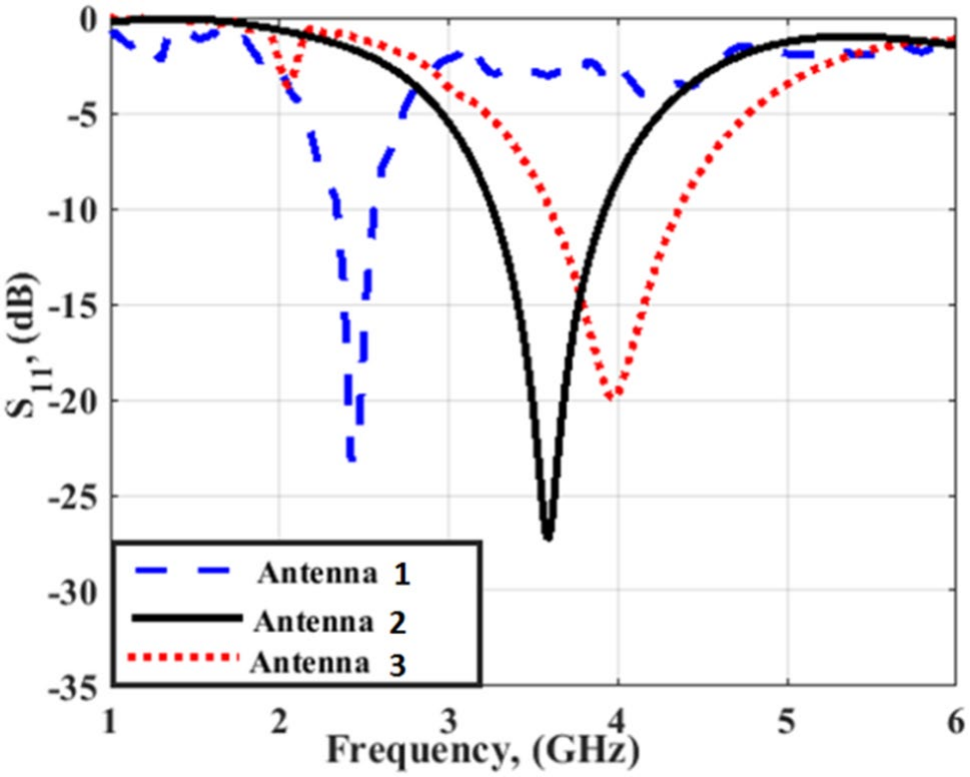
S_11_ of the three operation modes of reconfigurable antenna.

**Figure 5:**
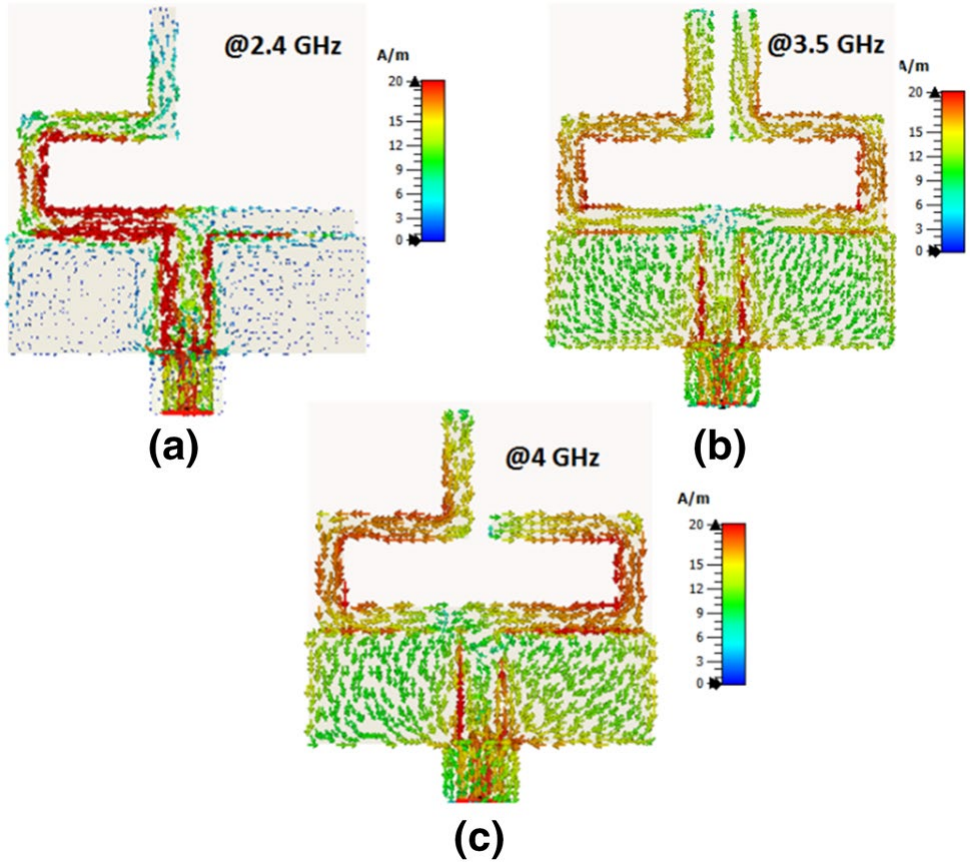
Simulated current distribution of the reconfigurable antenna at the three operating frequencies.

The antenna's performance is investigated in two different positions; flat and bent. The S_11_ of the reconfigurable flat antenna in the three modes is illustrated in Fig. 4. Based on the antenna development shown in Fig. 2, the Antenna 1 configuration is matched at 2.4 GHz while Antenna 2 configuration is matched at 3.5 GHz. Antenna 3 configuration shows good impedance matching at 4 GHz. Fig. 5 investigates the current distribution outcomes from the antenna at the operating frequency bands which shows the concentration of the surface currents around the arms at different operated frequency bands.

**Figure 6:**
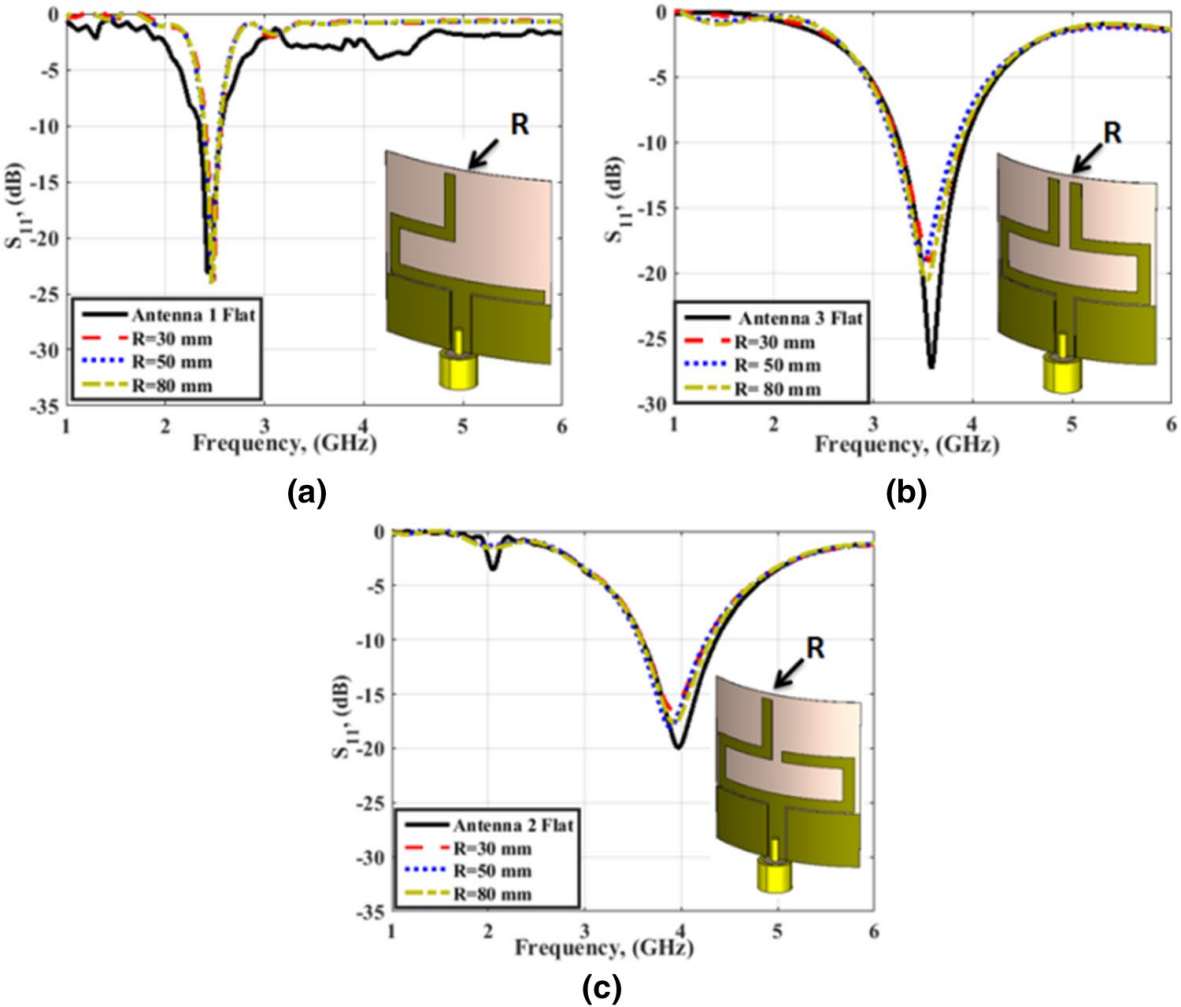
Antenna S_11_ at different degrees of bending in the three modes of operation.

To study the behavior of the antenna under bending, the antenna is bent around the y-axis as shown in Fig. 6. The antenna's S_11_ has been simulated at different degrees of bending for each mode of operation. Simulation results are presented in Fig. 6a–c. At each mode of operation, the antenna has been simulated at the bending of the radius of curvature R = 30 mm, 50 mm, and 80 mm. In all cases, the antenna's behavior remains stable and the effect of the bending on the return loss results is negligible.

### B. Performance of the antenna under reconfiguration

**Figure 7:**
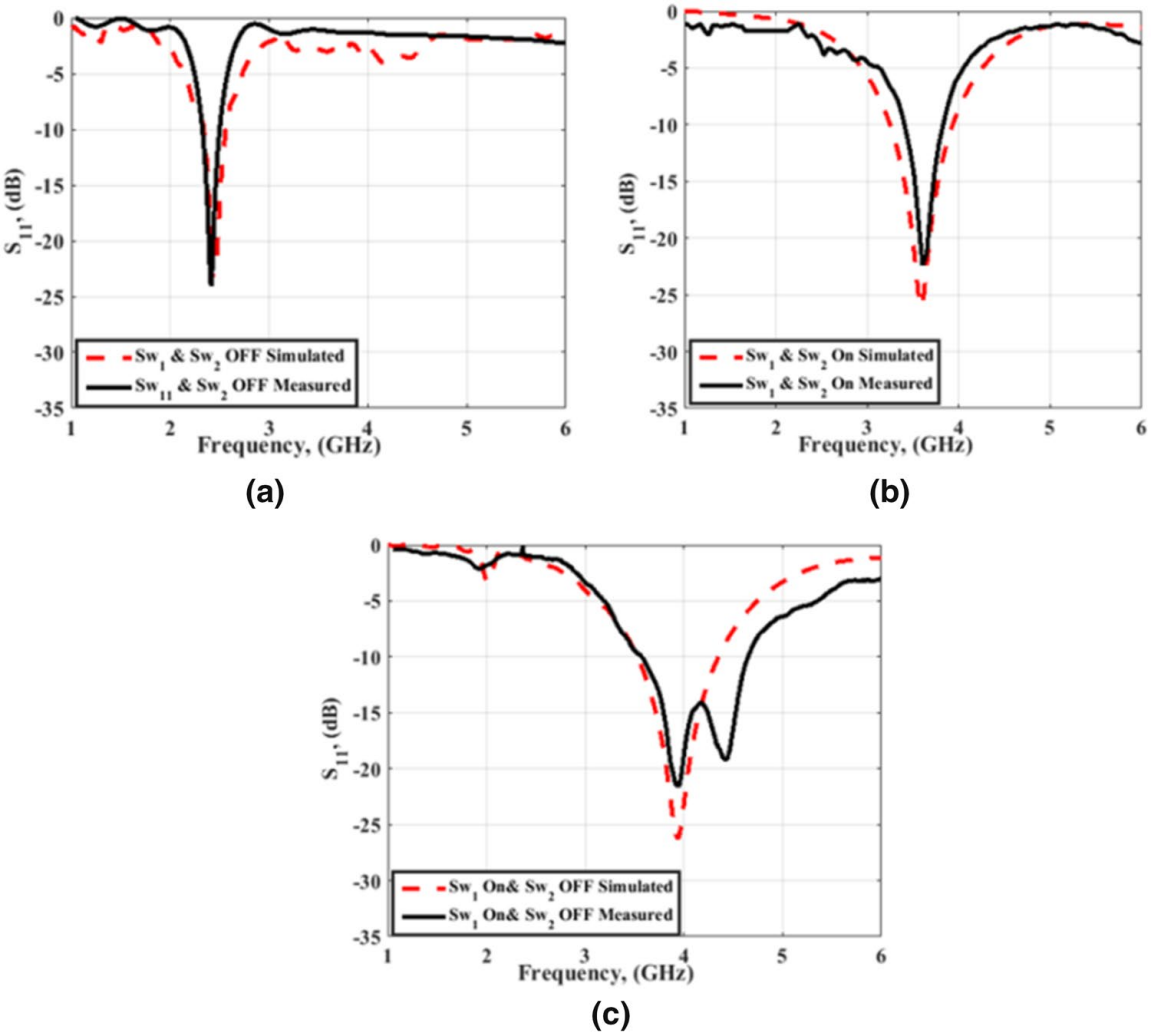
Simulated and tested S_11_of the antenna at the three modes of operation; (**a**) Sw1 OFF, Sw2 OFF (**b**) Sw1 ON Sw2 ON (**c**) Sw1 ON, Sw2 OFF.

After the fabrication, reconfiguration of the proposed antenna is done by controlling the two PIN diodes (HPND-4005), i.e., PIN#1 and PIN#2, which are connected through the two slots. The two PIN diodes are used as switches and controlled by the biasing voltages applied through the connecting wires as displayed in Fig. 3. The simulated and tested S_11_ curves of the reconfigurable antenna at the different modes of operation are displayed in Fig. 7. In the first mode, the two PIN diodes are biased such that an OFF state is obtained for the two diodes. In this case, the antenna is configured as an Antenna 1 with matching at 2.4 GHz, and the bandwidth is extended from 2.32 to 2.5 GHz (0.18 GHz). The simulated and tested S_11_ of the antenna in the first mode of operation is shown in Fig. 7a. There is a good match between the simulation and measurement outcomes. The second mode of operation is obtained by putting PIN#1 and PIN#2 in the ON state. Antenna 2 has resulted with good impedance matching at 3.5 GHz and the operating bandwidth is extended from 3.38 to 3.83 GHz (0.55 GHz). A good trend between the simulation and tested curves can be noticed as illustrated in Fig. 7b. The antenna's S_11_ with PIN#1 diode ON and PIN#2 OFF is illustrated in Fig. 7c. The antenna is matched at 4 GHz with an agreement between measurement and simulation results except for an additional resonance at 4.5 GHz in the case of the measurement curve. This is may due to the cables, the very thin substrates, measurement tolerances, and the switching feature of the PIN diodes. The operating bandwidth is extended from 3.5 to 4.5 GHz (1 GHz).

**Figure 8:**
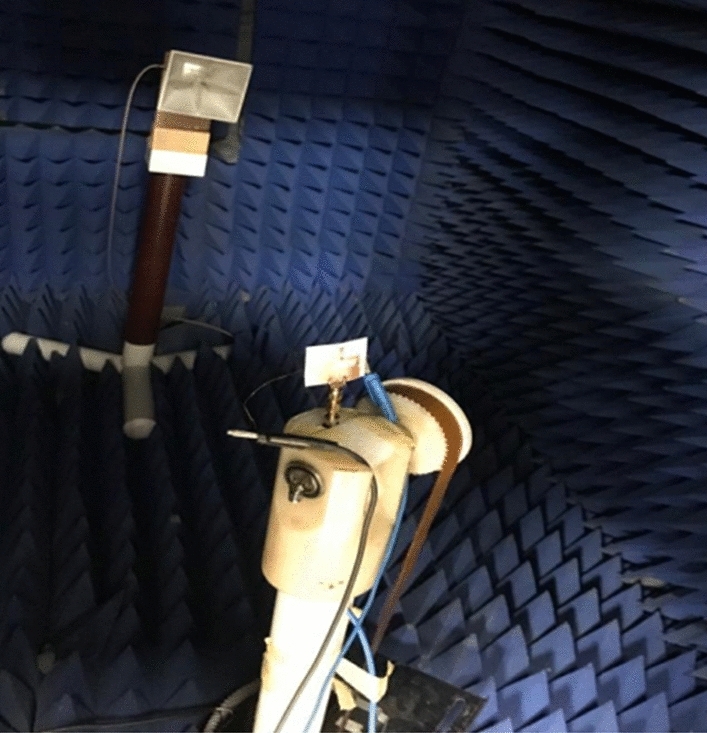
The radiation pattern testing setup.

**Figure 9:**
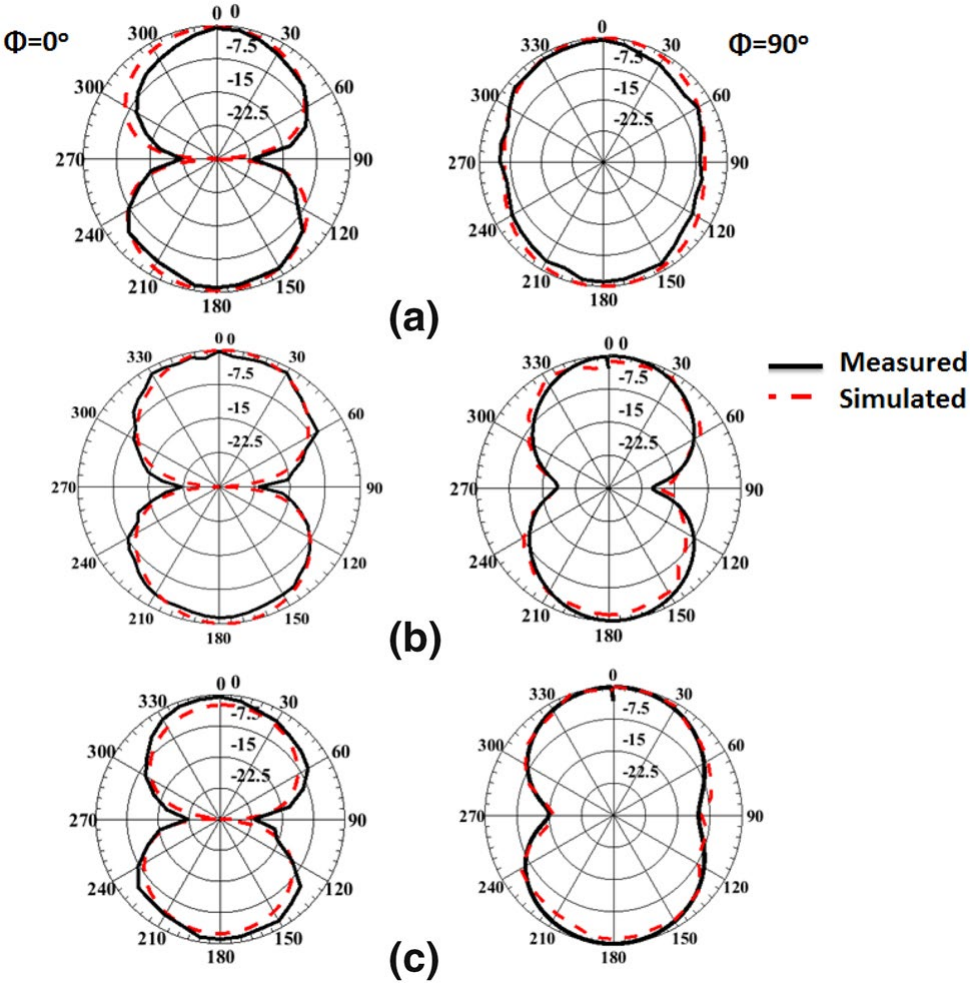
The radiation pattern simulated and tested in φ = 0° (LHS) and φ = 90° (RHS) at the three operating frequency bands; (**a**) 2.4 GHz, (**b**) 3.5 GHz, (**c**) 4 GHz.

The anechoic chamber is utilized to measure and extract the antenna's radiation pattern as displayed in Fig. 8. The radiation pattern is tested while the biasing of the PIN diodes is adjusted for each case during the measurement process. Fig. 9 displays the antenna's radiation pattern outcomes. The measurement results appear in a solid line while the simulation results are in a dashed line. The left-hand side (LHS) shows the pattern in the plane φ = 0° and the right-hand side (RHS) shows the φ = 90° plane pattern. As displayed in the figures, the radiation patterns have bidirectional outcomes at both planes with good trends between the two simulated and tested outcomes.

## Flexible reconfigurable antenna with FSS

**Figure 10:**
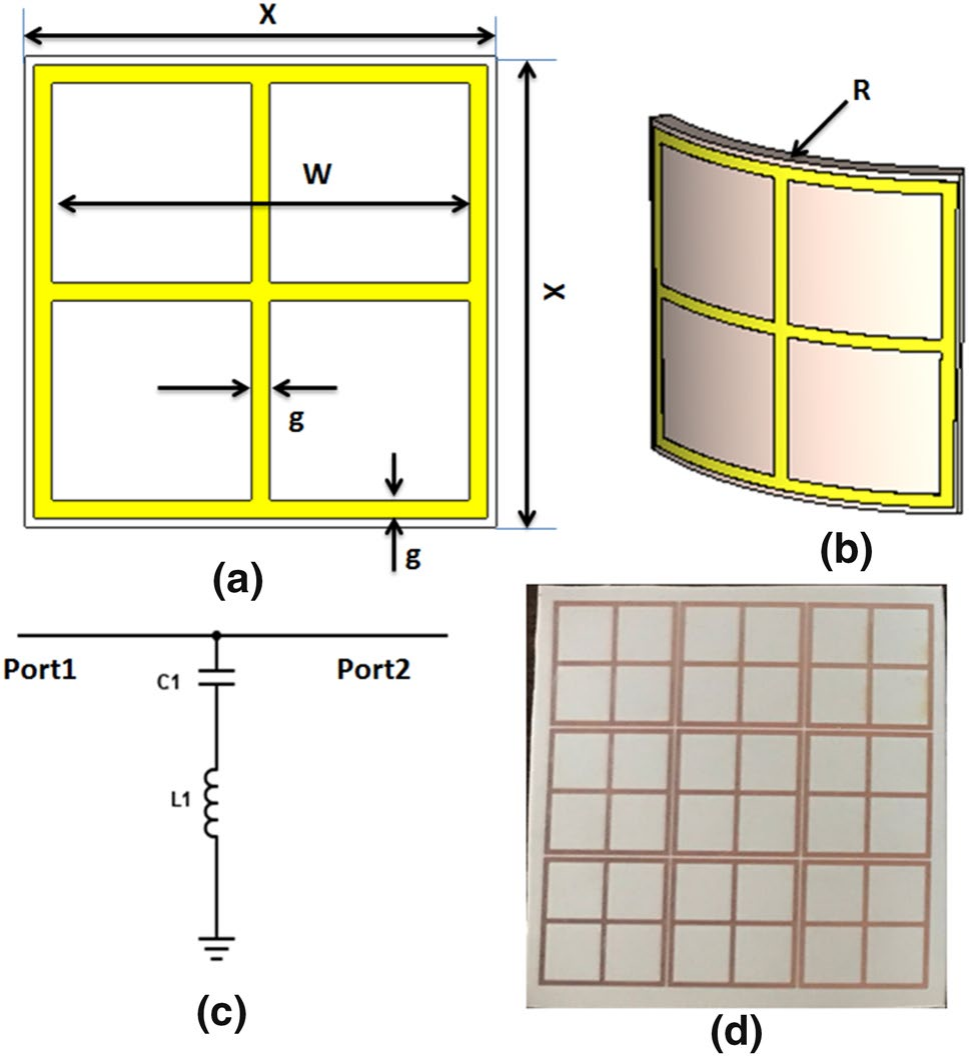
FSS model; (**a**) FSS unit element, (**b**) bending model of the FSS unit element at R = 50 mm, (**c**) FSS unit equivalent circuit, (**d**) complete FSS structure.

**Figure 11:**
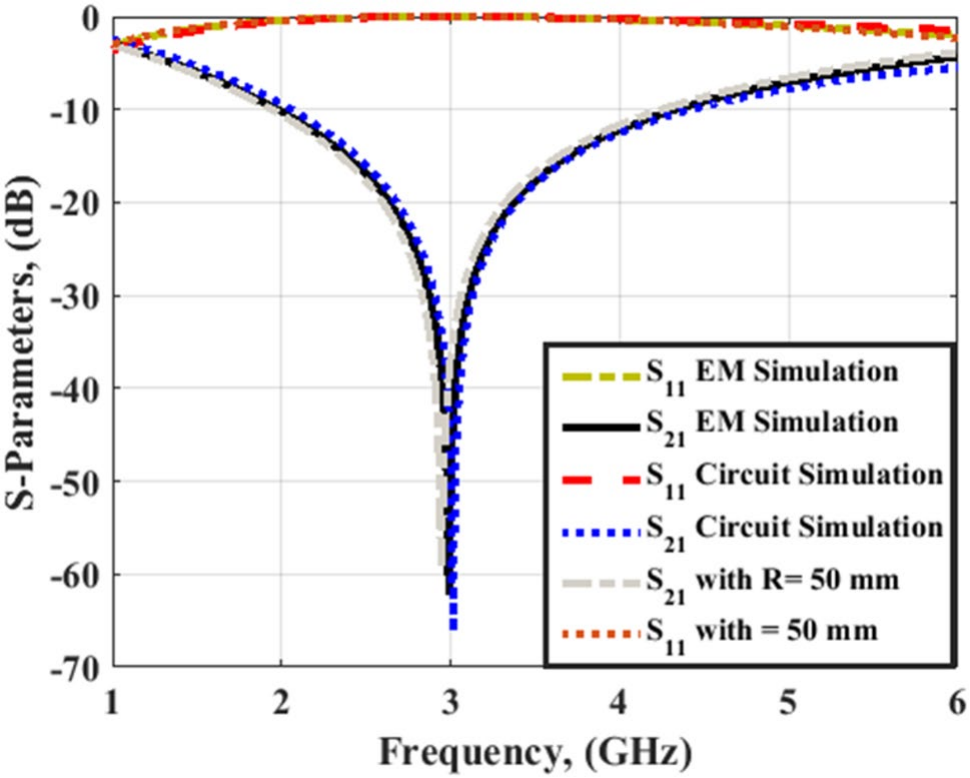
Simulated S-parameters of the FSS unit element.

It is highly required for the antenna to have a high gain. In this paper, an FSS is designed to improve the gain of the proposed antenna. The FSS is to be inserted under the antenna to force the radiated power in an upward direction. The FSS is a printed periodic structure with frequency filtering characteristics. The single unit of the proposed structure, as shown in Fig. 10a, is composed of a squared frame of four squared cells inside. Dimensions of the FSS unit cell are x = 26 mm, g = 1 mm, thickness of 1.57 mm, and w = 23 mm. The single FSS unit with R = 50 mm bending configuration is illustrated in Fig.10b. The electrical equivalent circuit of the FSS element is shown in Fig. 10c. The electrical equivalent circuit is a series inductor-capacitor branch that provides a band-stop filtering effect between Port 1 and Port 2. The equivalent values of the capacitor and inductor are C_1_ = 0.6587 pF and L_1_ = 4.328 nH. This behavior is utilized to enforce the reflection of the RF frequencies within the operating band of the reconfigurable antenna. The complete FSS structure is shown in Fig. 10d. Figure 11 illustrates the FSS unit cell S-parameters.

S_11_ is too high (approximately 0 dB) in the frequency band 2–4.5 GHz and S_21_ is too small (less than −10 dB) in the same frequency band in both flat and bending configurations. These results demonstrate the high reflectivity and low transmission through the proposed FSS structure. The best behavior of the FSS is at 3 GHz.

**Figure 12:**
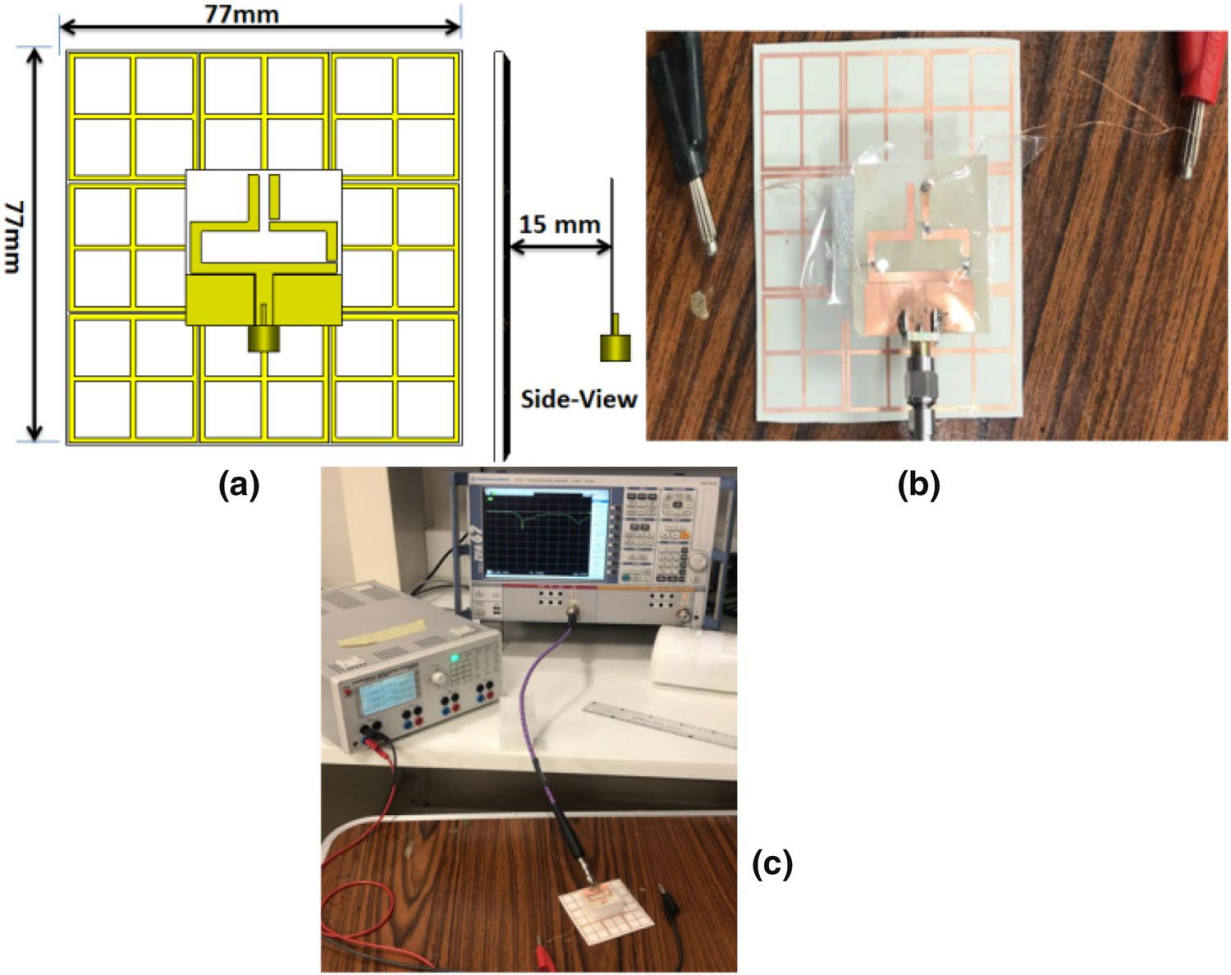
Simulation and testing setup of the antenna with FSS in a flat position; (**a**) top and side views of the antenna, (**b**) fabricated antenna spaced by a foam spacer, (**c**) testing setup.

The complete FSS is composed of 3 × 3 unit elements with 0.5 mm spacing between cells to achieve an overall dimension of 77 × 77 mm^2^ as seen in Fig. 12. The proposed FSS is put on ROGERS 3003 flexible substrate using the same technique of antenna fabrication. The fabricated FSS is tried with the reconfigurable antenna in two cases; flat and bent positions. In the case of a flat position, the suggested antenna is fixed above the FSS with a 15 mm foam spacer between them. Fig 12a illustrates the top and side views of the antenna with FSS. The distance of 15 mm is the optimized distance to achieve the desired matching and gain at the desired frequency bands. The fabricated antenna and FSS with foam spacer in between are displayed in Fig. 12b. The return loss of the flat antenna with FSS has been measured using VNA at the three frequencies 2.4 GH, 3.5 GHz, and 4 GHz. Measurement setup of the flat reconfigurable antenna with FSS is illustrated in Fig. 12c.

**Figure 13:**
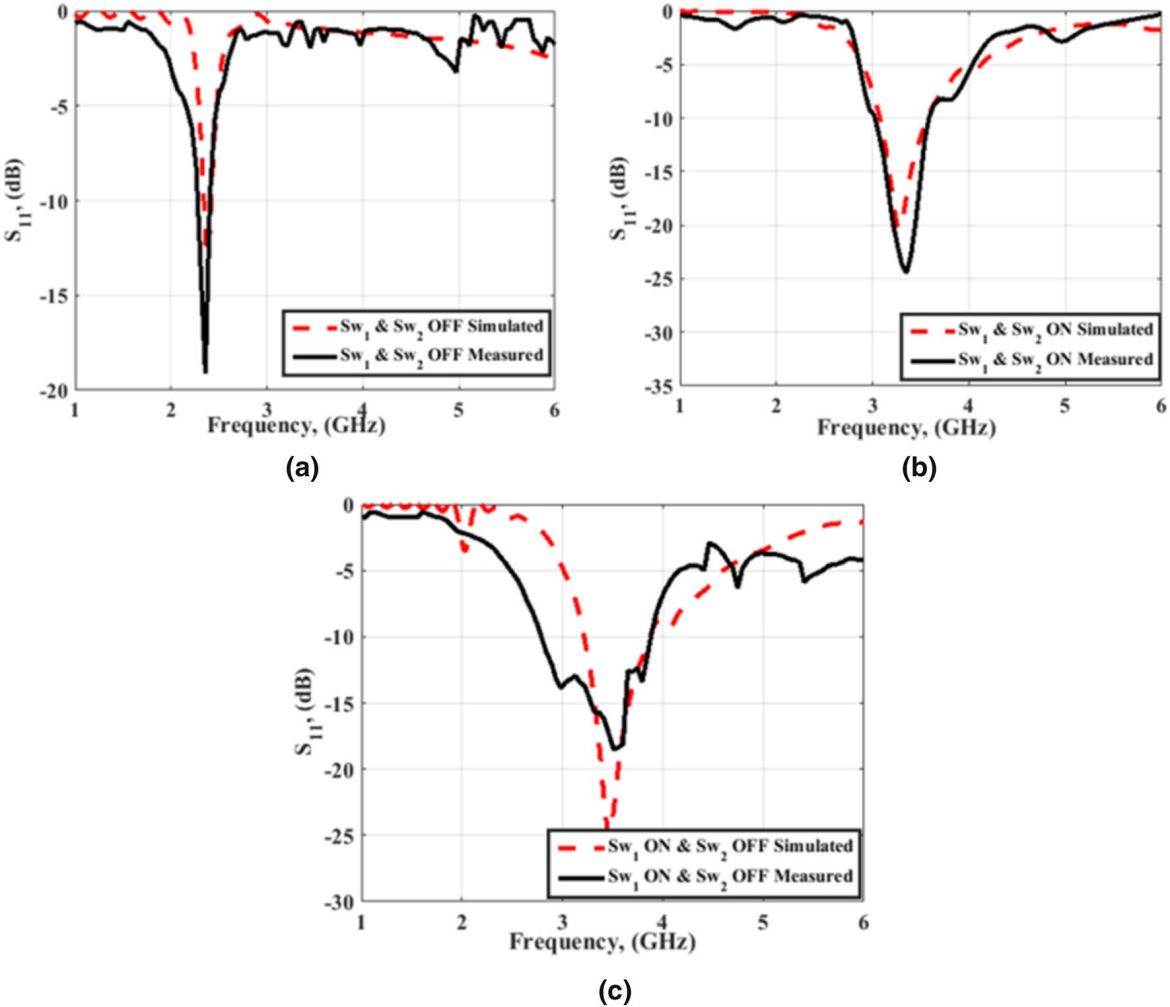
Simulated and tested S_11_ of the antenna with FSS in a flat position; (**a**) Sw1 OFF, Sw2 OFF (**b**) Sw1 ON, Sw2 ON (**c**) Sw1 ON, Sw2 OFF.

**Figure 14:**
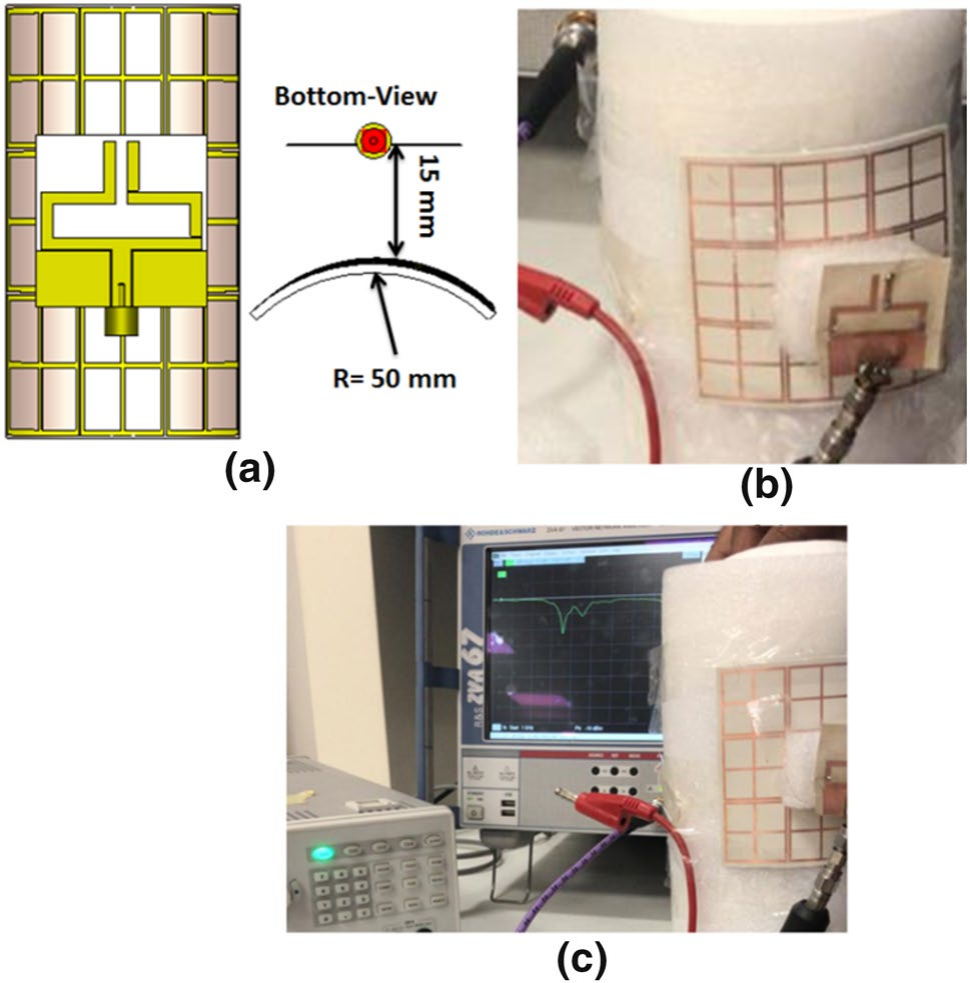
Simulation and tested setup of the reconfigurable antenna with FSS in a bent position; (**a**) top and side views, (**b**) fabricated antenna with the FSS supported by curved foam, (**c**) testing setup in a bent position.

The simulation and tested S_11_ of the flat reconfigurable antenna with FSS are shown in Fig. 13. The return loss of the antenna in the first mode of operation is displayed in Fig. 13a while the two PIN diodes are in the OFF state. The antenna is properly matched with S_11_ of −19 dB at the 2.4 GHz frequency. The return loss of the antenna with FSS in the second mode of operation while the two PIN diodes are ON is displayed in Fig. 13b. The antenna is matched over the frequency band 3 GHz to 3.6 GHz. The antenna's return loss in the third mode while the first diode is ON and the other is OFF is shown in Fig. 13c. It is seen that the antenna is matched from 2.8 GHz to 3.9 GHz. From the outcomes, it is noticed that the antenna's electrical behavior is affected by the existence of the FSS. By comparing with the behavior without FSS, the main resonance frequency of the antenna is shifted downward while the antenna still provides a wider frequency band in the case of measurement results. In addition to testing the reconfigurable antenna in a flat position, the antenna has been tested with FSS bending. The FSS is bent with a radius of curvature of 50 mm around the y-axis and placed under the antenna with 15 mm spacing as illustrated in Fig. 14. The simulation setup of the antenna with FSS is shown in Fig. 14a, and then the testing setup of Fig. 14b is constructed. In the testing setup, the FSS is bent by a 50 mm radius of curvature around the y-axis and is fixed in its position using a curved foam surface. The fabricated reconfigurable antenna is placed on the top of the curved FSS at a distance of 15 mm and fixed in its position using a foam cube in between them. The testing setup of the antenna with curved FSS is displayed in Fig. 14c where the DC power supply is used for the biasing of the PIN diodes and the VNA is used for the return loss measurements.

**Figure 15:**
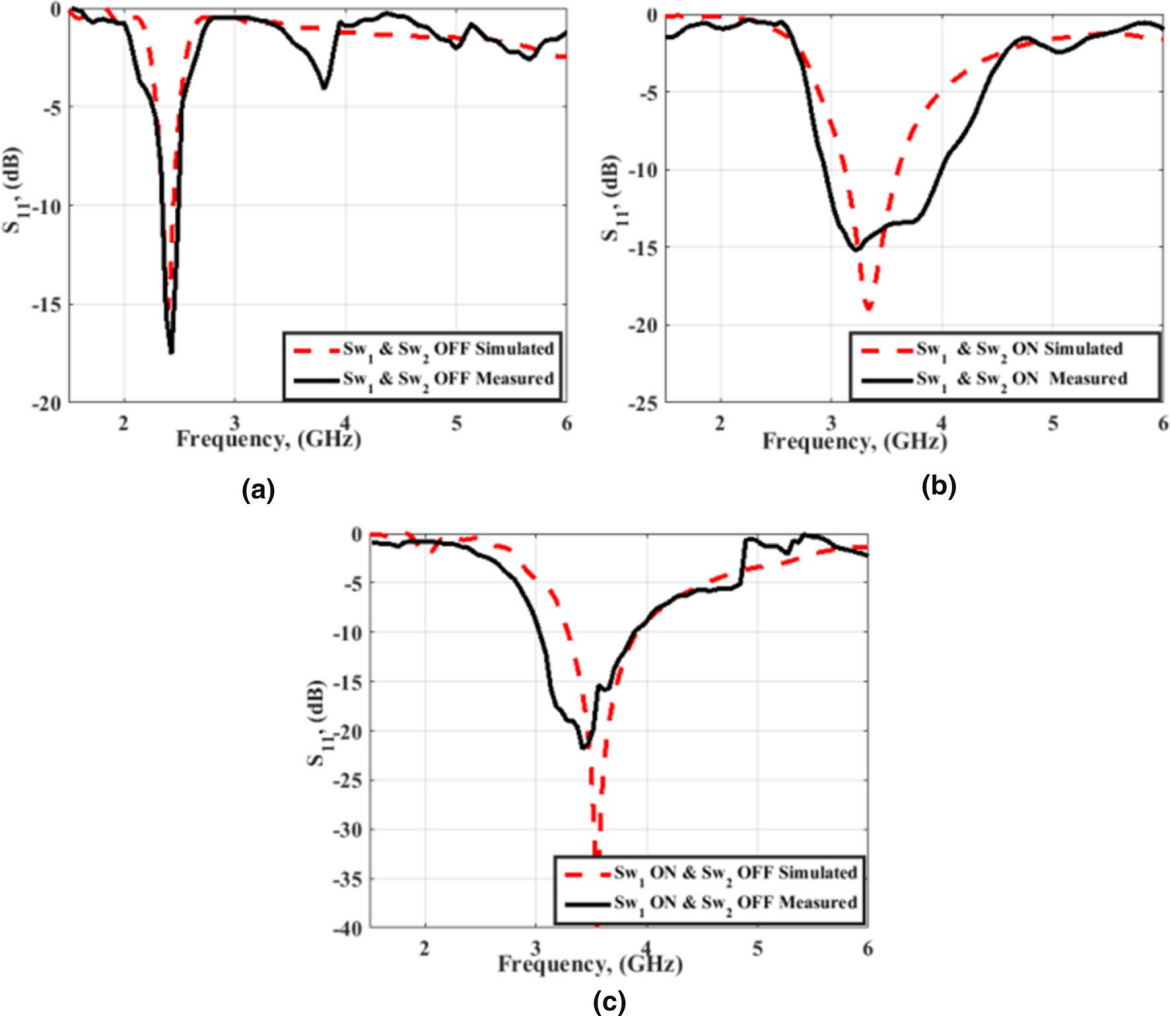
Simulated and tested S_11_ of the antenna with FSS in a b position; (**a**) Sw1 OFF, Sw2 OFF (**b**) Sw1 ON, Sw2 ON (**c**) Sw1 ON, Sw2 OFF.

**Figure 16:**
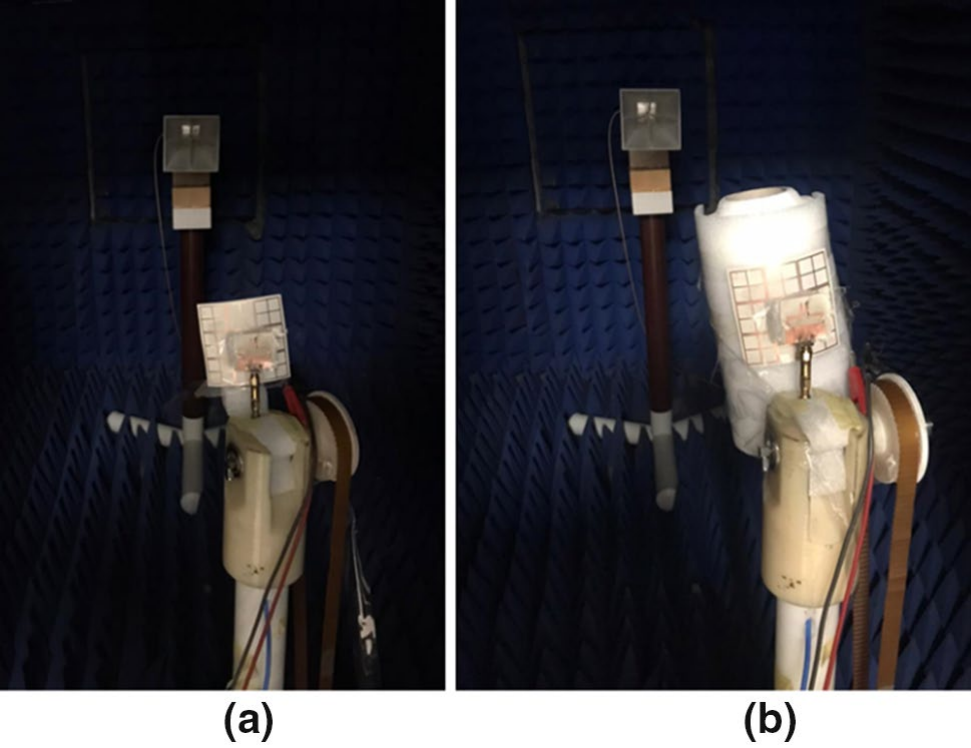
Testing setup of the antenna's radiation pattern (**a**) flat FSS, and (**b**) bent FSS.

**Figure 17:**
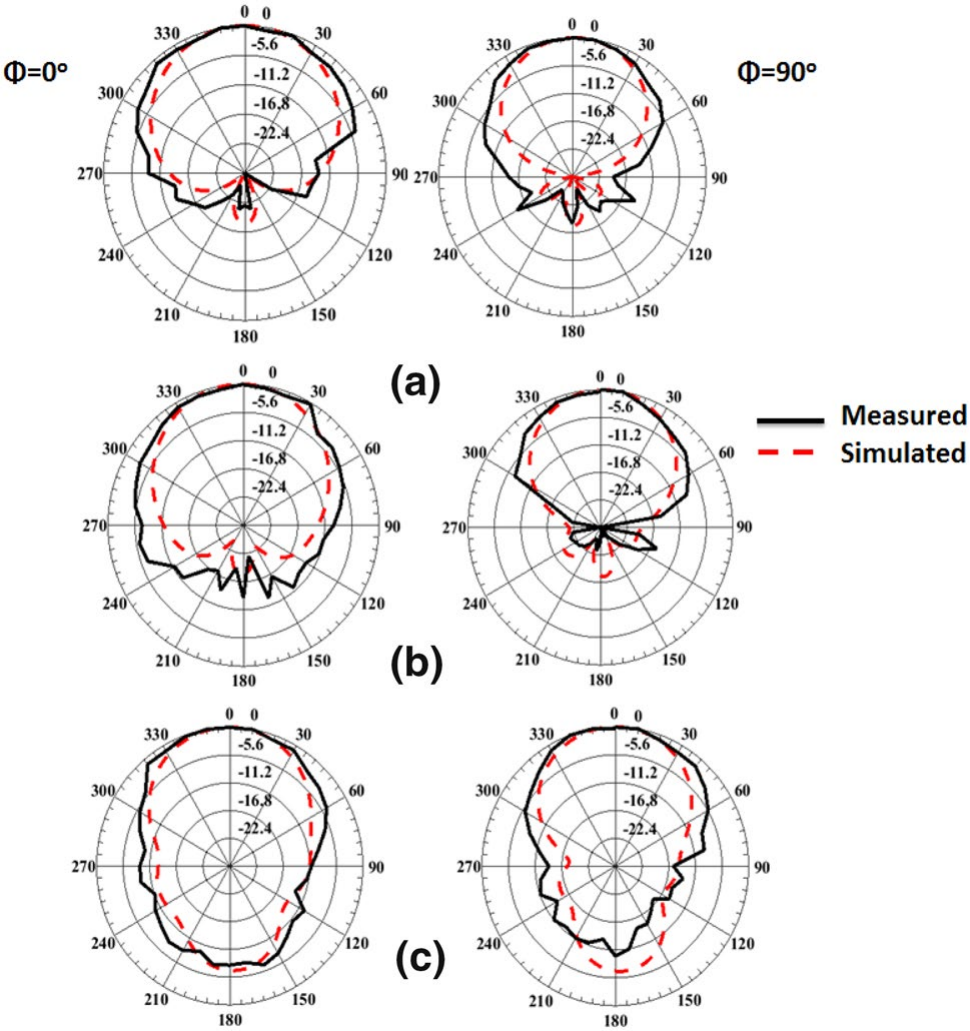
Simulated and measured radiation pattern of the reconfigurable antenna with FSS in φ = 0° (LHS) and φ = 90° (RHS) at the three operating frequency bands; (**a**) 2.4 GHz, (**b**) 3.5 GHz, (**c**) 4 GHz.

Looking at Fig. 15, we can see the simulated and tested return loss with curved FSS. The return loss of the antenna at mode 1 where the two PIN diodes are OFF is shown in Fig. 15a. The antenna is properly matched at 2.4 GHz. Switching the two PIN diodes into the ON state translates the resonance frequency to 3.5 GHz. The S_11_ of the antenna is less than -10 dB in the frequency band 3 GHz to 4 GHz as shown in Fig. 15b. By switching PIN#2 to the OFF state with maintaining PIN#1 at the ON state, the third mode of operation is obtained. In this case, the antenna is matched over the frequency band 3 GHz to about 4 GHz as shown in Fig. 15c. Although the existence of the FSS has affected the matching characteristics of the antenna, bending of the FSS does not affect the antenna's matching seriously. The observed deviation between the tested and simulation results is due to the cables, the very thin substrates, and measurement tolerances. To demonstrate the usefulness of using the FSS with the antenna, the gain outcomes have been tested as illustrated in Fig. 16. Figure 17 displays the radiation pattern outcomes with FSS. The LHS shows the pattern in the plane φ = 0° while the RHS shows the pattern in φ = 90° plane. Fig. 17a–c show the radiation pattern of the antenna. It is clearly shown that using the FSS with a reconfigurable antenna has directed the radiation pattern of the antenna upwards at the three modes of operation, and then a serious improvement in the antenna's gain has been achieved. Table 1 summarizes the simulated and measured gain of the antenna at the three modes of operation.

**Table 1:** Measured and simulated gain of the suggested antenna at the different modes of operation.

Antenna	Gain (dBi)					
	Sw1 & Sw2 OFF @ 2.4 GHz		Sw1 & Sw2 ON @ 3.5 GHz		Sw1 ON & Sw2 OFF @ 4 GHz	
	Meas	Sim	Meas	Sim	Meas	Sim
Without FSS (flat)	1.55	1.76	2.95	3.1	2.9	3.2
Without FSS (bent)	1.48	1.7	2.81	2.93	2.86	3.15
With FSS (flat)	6.5	7.15	7.52	8.2	7.91	8.5
With FSS (bent)	6.21	6.51	7.23	7.53	7.36	7.7

According to the measurements of the antenna with and without FSS, the antenna's gain in the presence of the FSS has been improved as follows: from 1.55 dBi (without FSS) to 6.5 dBi (with FSS) at 2.4 GHz, from 2.95 to 7.23 dBi at 3.5 GHz, and from 2.9 to 7.91 dBi at 4 GHz. The proposed antenna outcomes in comparison with other designs are summarized in Table 2.

**Table 2:** Comparison between the suggested Antenna and other designs.

Ref	Antenna size mm^3^/(λ_o_^3^)	Reconfiguration type	Frequency bands (GHz)	Antenna type	No. of switches	Gain (dBi)	Applications	Flexibility
^6^	25 × 25 × 1.6 (0.32λo × 0.32λo × 0.02λo)	Frequency	3.85/4.14/4.43/4.91/6.01	Rectangular slotted with defected ground	3 PIN diodes	2/2.9/2.5/4.42/3.01	IoT, WLAN, Wi-Max, and C-band	No
^7^	40 × 40 × 1.5 (0.18λo × 0.18λo × 0.007λo)	Frequency	Continuous tuning (1.4–2.9)	Hexagonal patch element	1 Varactor diode	Max. 2.4	IoT, WiFi, Bluetooth,	No
^13^	32 × 25 × 0.254 (0.19λo × 0.15λo × 0.001λo)	Frequency	1.8/1.9/2.1/2.45/3.5	Quarter-wave monopole with filtering stub	3 PIN diodes	2.34/2.74/3.02/3.12/3.2	GSM, ISM, and 5G	Yes
^17^	35 × 25 × 0.254 (0.28λo × 0.20λo × 0.002λo)	Frequency	(2.76–8.21)/2.45/5.8/5.2/8	CPW-fed slotted circular patch	1 PIN diode	Min. 2.49/max. 5.8	ISM, WLAN, and UWB bands	Yes
^20^	40 × 50 × 0.254 (0.22λo × 0.27λo × 0.001λo)	Frequency and radiation pattern	(1.65–2.5)/1.8/2.1	A triangular monopole	2 PIN diodes	Max. 2.2	GSM, ISM, cellular communications, long-term evolution (LTE) bands	Yes
^29^	20 × 20 × 0.8 (0.21λo × 0.21λo × 0.008λo)	Frequency	3.22/4.99/7.35/7.26	Slot-based square-shaped monopole	1 PIN diode	2.5/2.7/3.8/2	Bluetooth, WiMAX, and upper WLAN	Yes
^30^	80 × 80 × 0.79 (0.46λo × 0.46λo × 0.004λo)	Frequency	1.76/5.71	Slot-ring antenna	16 PIN diodes	0.1/4.2	L-band and C-band	No
This work	30 × 30 × 0.13 (0.24λo × 0.24λo × 0.001λo)	Frequency	2.4/3.5/4	CPW-fed monopole	2 PIN diodes	1.55/2.95/2.9	IoT, Bluetooth, ZigBee, Wifi, WiMax, and C-band	Yes
	77 × 77 × 15 (0.61λo × 0.61λo × 0.12λo)	Frequency	2.4/3.5/4	CPW-fed monopole	2 PIN diodes	6.5/7.52 / 7.91	IoT, Bluetooth, ZigBee, Wifi, WiMax, and C-band	Yes

## Conclusion

A frequency-reconfigurable antenna with flexible characteristics equipped with an FSS structure is presented in this paper. The antenna is intended for IoT applications and switches between three frequency bands; 2.4 GHz, 3.5 GHz, and 4 GHz. The antenna has been simulated, fabricated, and measured to investigate its performance. Good electrical characteristics have been obtained at the three bands of operation. The performance of the antenna under bending has been tested while stable behavior has been obtained at different degrees of bending. In addition to the reconfigurability and flexibility, the antenna is equipped with FSS to improve its gain. The performance of the flexible reconfigurable antenna with FSS has been validated using simulations and measurements. The obtained results show that the FSS has contributed to a high improvement in the antenna's gain. All of the measurement results have good matching with the simulated outcomes which supports the suggested antenna to be utilized in IoT systems. 

## Data Availability

All data generated or analyzed during this study are included in this article (and there are no supplementary materials).
